# *COL7A1* Editing via RNA *Trans*-Splicing in RDEB-Derived Skin Equivalents

**DOI:** 10.3390/ijms24054341

**Published:** 2023-02-22

**Authors:** Bernadette Liemberger, Johannes Bischof, Michael Ablinger, Stefan Hainzl, Eva M. Murauer, Nina Lackner, Patricia Ebner, Thomas Kocher, Alexander Nyström, Verena Wally, Elisabeth Mayr, Christina Guttmann-Gruber, Josefina Piñón Hofbauer, Johann W. Bauer, Ulrich Koller

**Affiliations:** 1 EB House Austria, Research Program for Molecular Therapy of Genodermatoses, Department of Dermatology and Allergology, University Hospital of the Paracelsus Medical University, 5020 Salzburg, Austria; 2 Department of Dermatology, Medical Faculty, Medical Center—University of Freiburg, 79110 Freiburg, Germany; 3 Department of Dermatology and Allergology, University Hospital of the Paracelsus Medical University, 5020 Salzburg, Austria

**Keywords:** *COL7A1*, dystrophic epidermolysis bullosa, RNA therapy, RNA *trans*-splicing

## Abstract

Mutations in the *COL7A1* gene lead to malfunction, reduction or complete absence of type VII collagen (C7) in the skin’s basement membrane zone (BMZ), impairing skin integrity. In epidermolysis bullosa (EB), more than 800 mutations in *COL7A1* have been reported, leading to the dystrophic form of EB (DEB), a severe and rare skin blistering disease associated with a high risk of developing an aggressive form of squamous cell carcinoma. Here, we leveraged a previously described 3′-RTMS6m repair molecule to develop a non-viral, non-invasive and efficient RNA therapy to correct mutations within *COL7A1* via spliceosome-mediated RNA *trans*-splicing (SMaRT). RTM-S6m, cloned into a non-viral minicircle-GFP vector, is capable of correcting all mutations occurring between exon 65 and exon 118 of *COL7A1* via SMaRT. Transfection of the RTM into recessive dystrophic EB (RDEB) keratinocytes resulted in a *trans*-splicing efficiency of ~1.5% in keratinocytes and ~0.6% in fibroblasts, as confirmed on mRNA level via next-generation sequencing (NGS). Full-length C7 protein expression was primarily confirmed in vitro via immunofluorescence (IF) staining and Western blot analysis of transfected cells. Additionally, we complexed 3′-RTMS6m with a DDC642 liposomal carrier to deliver the RTM topically onto RDEB skin equivalents and were subsequently able to detect an accumulation of restored C7 within the basement membrane zone (BMZ). In summary, we transiently corrected *COL7A1* mutations in vitro in RDEB keratinocytes and skin equivalents derived from RDEB keratinocytes and fibroblasts using a non-viral 3′-RTMS6m repair molecule.

## 1. Introduction

Epidermolysis bullosa (EB) is a rare, monogenetic blistering disease caused by mutations within genes responsible for the expression of structural proteins in the skin. One of the affected genes is *COL7A1*, in which mutations lead to the recessively-inherited dystrophic EB subtype (RDEB) [1]. *COL7A1* codes for type VII collagen (C7), an extracellular matrix protein expressed by epidermal keratinocytes and dermal fibroblasts in the basement membrane zone (BMZ) of the skin. The helical C7 trimer, with a size of 900 kDa, constitutes the main component of anchoring fibrils and hence is necessary for the structural integrity of the skin [2]. Due to indels, nonsense or splice site mutations, C7 protein expression is either reduced or completely absent in RDEB skin, causing impaired adhesion between the dermal and epidermal layers. As such, a minor mechanical impact can lead to blistering and/or erosions of the skin and mucous membranes [1]. Aside from severe pain, patients with RDEB are prone to develop chronic wounds and have a higher risk of developing a particularly aggressive, metastasising form of squamous cell carcinoma that is associated with a lower life expectancy [3,4]. Mutations in *COL7A1* are not limited to certain hotspots but can occur anywhere along the entire length of the gene, which spans 118 exons [5]. The resulting *COL7A1* transcript is 9.2 kb in length, beyond the packaging capacity of many delivery vectors. Spliceosome-mediated RNA *trans*-splicing (SMaRT) is an established technology able to correct multiple mutations with only one repair molecule, the RNA *trans*-splicing molecule (RTM) [6,7,8]. To induce a *trans*-splicing reaction, the RTM is delivered into the cell’s nucleus to bind the target region via a very specific binding domain (BD). The RTM additionally carries splicing elements and the portion of the wild-type *COL7A1* cDNA sequence that will replace the mutation-harbouring region of the transcript. *Trans*-splicing can be employed to replace the 5′ region or the 3′ regions of the transcript (referred to as 5′ *trans*-splicing and 3′ *trans*-splicing, respectively) or an internal region of the mRNA (internal exon replacement, IER) [9,10]. Therefore, depending on the gene positions of the mutations to be targeted, different RTMs can be designed to encode only the corresponding wild-type regions of the gene needed. The reduced transgene size (in comparison to the introduction of the full-length cDNA), the maintenance of endogenous control of gene expression (via the gene’s natural promoter), and the ability to simultaneously reduce the levels of mutated transcripts are but a few advantages this technology provides [11]. Hence, SMaRT has already been successfully applied to correct mutations in various diseases such as muscular dystrophy [12], spinal muscular atrophy [13,14], severe combined immune deficiency [15] and cystic fibrosis [16]. In EB, the functionality of *trans-*splicing has been demonstrated for various mutations in *COL17A1* [6], *PLEC* [17], *KRT14* [11,18,19] and *COL7A1* [20,21,22].

In a previous study, a functional 3′ RTM had been designed to replace a 3.3 kb sequence of the *COL7A1* transcript [21], covering approximately 40% of all DEB-causing mutations between exon 65 and 118 [23]. The 3′ RTM was expressed in a lentiviral vector and has been shown to functionally correct the RDEB phenotype in keratinocytes and skin equivalents in vitro via *trans-*splicing correction of the human *COL7A1* mRNA.

However, to mitigate potential safety concerns regarding the use of an integrating viral vector in clinical practice, we sought to develop a therapeutic approach based on a minicircle vector in a liposomal formulation to enable topical RTM delivery into RDEB keratinocytes and fibroblasts. Here, we detect correct *trans*-splicing on the mRNA level, as well as an increased C7 expression in RDEB cell lines and skin equivalents, showing the feasibility of this approach.

## 2. Results

### 2.1. In Vitro 3′ Trans-Splicing in RDEB Keratinocytes and Fibroblasts

**Figure 1:**
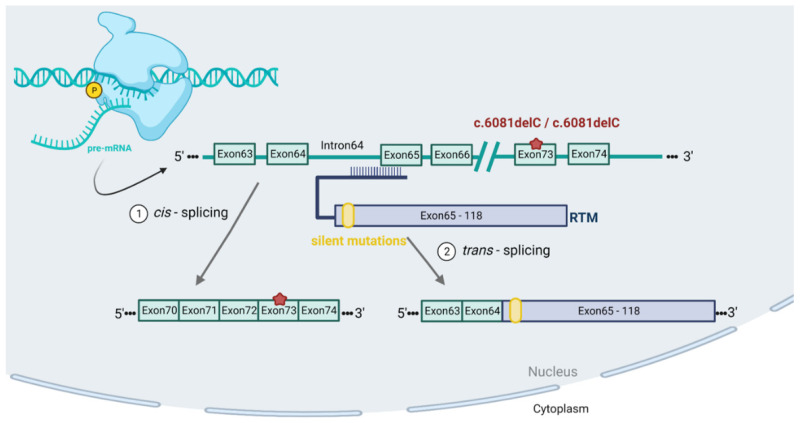
Schematic overview of the endogenous splicing processes. After the introduction of the RTM into the cell’s nucleus, the binding domain (BD) specifically targets the *COL7A1* pre-mRNA by binding the intron 64/exon 65 junction. By providing an alternative splice acceptor site, the RTM facilitates the replacement of the mutated gene region within the *COL7A1* pre-mRNA with the wild-type cDNA sequence (exon 65–exon 118) from the RTM, leading to a correction of the mutation. To enable an easy distinction between the *cis*-spliced and the *trans*-spliced pre-mRNA on the RNA level, the RTM carries five silent mutations within exon 65, which can be easily targeted with a polymorphism-specific reverse primer as recently described [21]. Created with BioRender.com (accessed on 1 February 2023).

To evaluate the functionality of the parental minicircle vector harbouring the RTMS6m, patient cells were transiently transfected with the MC-RTMS6m plasmid. Here, we used an immortalised RDEB keratinocyte cell line harbouring a homozygous frameshift mutation (c.6081delC/c.6081delC) within exon 73 of *COL7A1* [24] and immortalised RDEB fibroblasts carrying either mutation in exon 115 (c.8441-15del20/c.8505-8506dupCG) or exon 105 (c.7795G>T/c.7795G>T). After successful delivery into the cell’s nucleus, the RTM binds its target sequence on the *COL7A1* pre-mRNA (Figure 1) [21]. Therefore, the RTM contains a) a binding domain (BD) specifically binding the intron 64/exon 65 junction, b) an alternative splice site and c) the wild-type *COL7A1* cDNA sequence spanning from exon 65 to exon 118 to replace the corresponding region on the pre-mRNA. Upon correct *trans*-splicing with the mutated *COL7A1* pre-mRNA, a chimeric product derived from the *COL7A1* pre-mRNA (exon 1–exon 64) and the RTM (exon 65–exon 118) is generated. To facilitate the distinction between the endogenous *cis*-spliced version of the mutated *COL7A1* mRNA and the *trans*-spliced *COL7A1*/RTM chimaera, five silent mutations were introduced into the 5′ coding region of the RTM [21].

**Figure 2:**
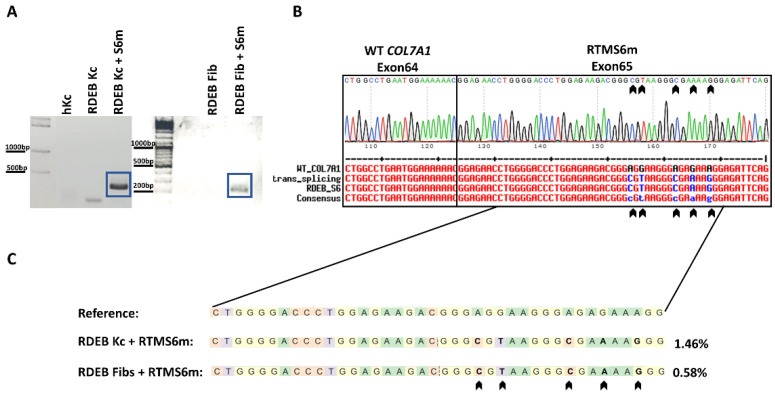
Endogenous *trans*-splicing in RDEB keratinocytes and fibroblasts. (**A**) Correctly *trans*-spliced mRNA products were detected after transfection with the RTMS6m minicircle vector via sqRT-PCR, using a *COL7A1*-specific forward primer and an RTM-specific reverse primer, targeting all five silent mutations (black arrows) introduced at the beginning of the RTM sequence. The *trans*-spliced mRNA product (blue boxes) has a size of 209 bp and was detected in RDEB keratinocytes and RDEB fibroblasts transfected with the MC-RTMS6m plasmid. Healthy keratinocytes (hKc) and untransfected RDEB keratinocytes and fibroblasts were used as negative controls. (**B**) Sanger sequencing of the PCR product showed all silent mutations introduced by the RTMS6m and hence confirmed the accuracy of the *trans*-splicing reaction. (**C**) Additionally, NGS analysis showed *trans*-splicing in both RDEB keratinocytes and fibroblasts, with an efficiency of ~1.5% and ~0.6%, using the same exon 61/exon 62 junction forward primer in combination with an exon 68 specific reverse primer. The wild-type *COL7A1* sequence was used as reference. Analysis of NGS results was performed with CRISPResso2 [25].

To detect the *trans*-splicing process on the mRNA level, semi-quantitative reverse transcription and polymerase chain reaction (sqRT-PCR) analysis was performed, using a *COL7A1*-specific forward primer targeting the exon 61/exon 62 junction and a reverse primer that specifically binds the silent mutations encoded by the RTM (Figure 2, black arrows). The resulting 209 bp product was detected only in RDEB keratinocytes treated with the MC-RTMS6m plasmid (Figure 2A). Sanger sequencing of the PCR product confirmed the accuracy of the *trans*-splicing reaction in the RTM-treated patient cells, showing a chimeric mRNA sequence of wild-type *COL7A1* with the incorporation of the silent mutations from the RTM (Figure 2B). To establish the efficiency of the RTM, NGS analysis was performed on transfected RDEB keratinocytes and fibroblasts and compared to untreated RDEB cells. Analysis of the NGS raw data was performed via CRISPRESSO 2.0 and showed a *trans*-splicing efficiency of ~1.5% in keratinocytes and ~0.6% in fibroblasts (Figure 2C) [25].

### 2.2. Minicircle RTMS6m Expression Induces C7 Restoration in RDEB Keratinocytes and Fibroblasts

**Figure 3:**
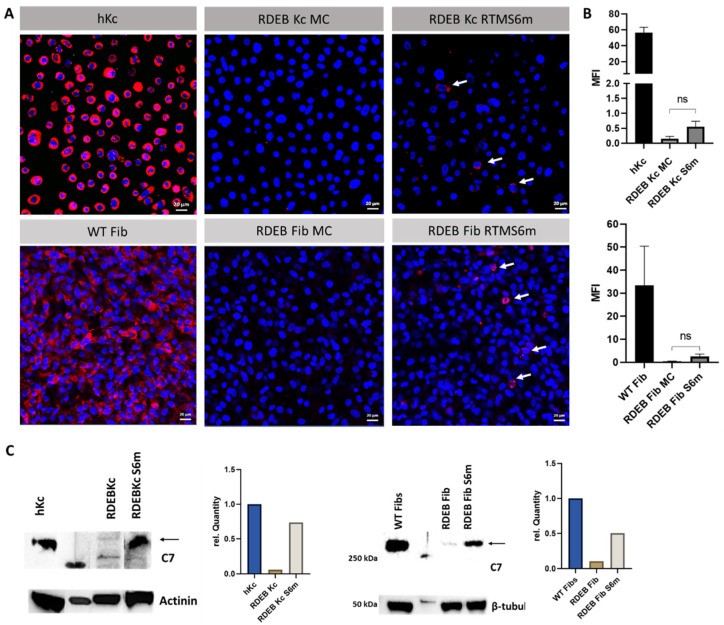
C7 expression in RDEB keratinocytes and fibroblasts. (**A**) Immunofluorescence staining of RDEB patient cell lines treated with the MC RTMS6m plasmid (right). RTM-treated patient keratinocytes, as well as fibroblasts, showed a restoration of C7 in individual cells (white arrows). Patient cell lines treated with the parental MC vector were used as negative controls (middle). Keratinocytes and fibroblasts from healthy donors served as a positive control (left). The cell’s nuclei were stained with 4′,6-Diamidino-2-phenylindol (DAPI, blue). Scale bar = 20 µm. (**B**) Mean fluorescence intensity was analysed with Fiji, and calculations were done using GraphPad Prism 9 (*n* = 3; mean ± SEM) [27,28]. (**C**) Expression of C7 after RTMS6m treatment of keratinocytes and fibroblasts was confirmed by Western blot analysis. Signal intensity was quantified using densitometric analysis with the Image Lab™ software from Biorad and plotted using GraphPad Prism 9.

To demonstrate the expression of *trans*-spliced C7 in MC RTMS6m-transfected RDEB keratinocytes and fibroblasts, we transfected cell monolayers and performed immunofluorescence staining using a human-specific anti-C7 antibody [26]. Both RDEB keratinocytes and RDEB fibroblasts showed a restoration of C7 in individual cells in comparison to keratinocytes and fibroblasts treated with the parental minicircle vector lacking the RTMS6 sequence (Figure 3A,B). Western blot analysis of RDEB keratinocytes and fibroblasts treated with the MC RTMS6m confirmed the enhanced expression of C7 compared to untreated patient cells (Figure 3C).

### 2.3. Accumulation of Restored C7 within Skin Equivalents

**Figure 4:**
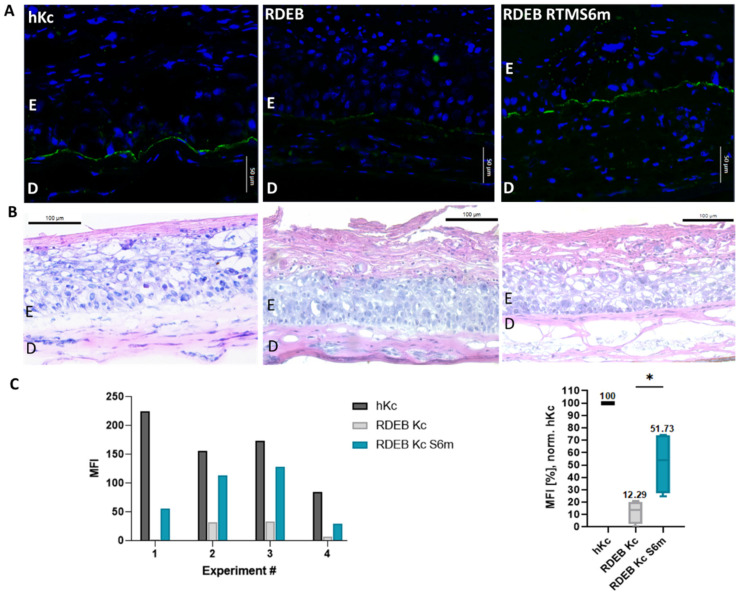
Correction of RDEB skin equivalents. (**A**) Immunofluorescence staining performed on cryosections showed enhanced C7 expression (green) in RDEB fibroblast/RDEB keratinocyte SEs topically treated with the RTMS6m (right) as compared to untreated RDEB/RDEB SE (middle). SEs from wild-type fibroblasts and healthy keratinocytes were used as a positive control (left). Cell nuclei were stained with 4′,6-Diamidino-2-phenylindol (DAPI, blue). Scale bars: 20 µm. E= Epidermis; D = Dermis (**B**) Formation and organisation of the SE were confirmed by H&E staining. Scale bars: 100 µm. (**C**) The Mean Fluorescence Intensity (MFI) of the C7 expression was analysed with Fiji [27,28]. Final MFI calculations were done by subtracting the background MFI from the basal layer fluorescence. Treatment with RTMS6m led to increased C7 expression in four independent experiments (left). Calculation of the mean MFI in reference to the hKc SE C7 expression showed a significant (*) 4-fold increase in treated vs. untreated RDEB SE (right). Significance was determined via an unpaired Student’s *t*-test (*n* = 4; *p* = 0.0272; mean ± SEM).

To verify functionality and correct localisation of restored C7, immortalised RDEB fibroblasts and RDEB keratinocytes were used to generate full-thickness skin equivalents (SE). For topical application, the MC RTMS6m-expressing plasmid was complexed in a 1:8 ratio with DDC642, a liposomal carrier [29]. The liposome/plasmid complex was applied dropwise onto the surface of the fully differentiated skin equivalents three times over the course of 1 week. Using a human C7-specific antibody, staining of cryosections from SEs treated with the minicircle RTMS6m complex showed a clear enhancement of the immunofluorescent signal correlating with the C7 expression (Figure 4A). The restored C7 was accurately located within the basement membrane zone, between the dermal and the epidermal layer. Haematoxylin and Eosin (H&E) staining was done to confirm the formation of a stratified epidermis, which was variable due to the use of immortalised cell lines (Figure 4B). Fiji analysis of mean fluorescence intensity (MFI) showed a 4-fold increase in SE treated with the MC RTMS6m (~52% MFI) in comparison to untreated RDEB SE (~12% MFI) (Figure 4C), in which traces of mutated C7 were detectable [27,28].

## 3. Discussion

So far, the treatment of DEB patients is mainly limited to daily wound care, continuous control visits and biopsies in at-risk patients to monitor the onset of cancer development. A causal therapy would greatly enhance the quality of life and general life expectancy of patients. Many promising results have been published using the CRISPR/Cas9 technology to efficiently correct mutations in epidermolysis bullosa, especially for mutations in *COL7A1* [24,30,31,32,33,34], *COL17A1* [35,36], *KRT5* [37] and *KRT14* [38]. However, gene editing via CRISPR/Cas9 is associated with some drawbacks, including possible off-target events, the requirement of an adjacent protospacer motif (PAM) near the site of mutation, as well as DNA-damage toxicity due to double-strand breaks (DSB) possibly triggering apoptosis [39,40]. Another therapeutic option, the retroviral transduction of full-length *COL7A1* cDNA into the genome of patient cells, bears the risk of genetic rearrangements of the transgene. One such recently-conducted phase I/II clinical trial showed improved wound healing and anchoring fibril formation at the graft sites. Nevertheless, C7 expression was significantly reduced over time [41,42]. Guide et al. recently presented the outcome of a phase 3 clinical trial using Beremagene Geperpavec (B-VEC) for dystrophic epidermolysis bullosa [43]. Here, a non-integrating and non-replicating herpes simplex virus type 1 (HSV-1) containing *COL7A1* was used to treat RDEB skin. Application of the gel over a time span of 26 weeks led to complete healing in 67% of the wounds exposed to B-VEC as compared with 22% of those exposed to placebo at month 6. In summary, the study showed that complete wound healing in patients with dystrophic epidermolysis bullosa was more presumable with topical administration of B-VEC than with a placebo. Due to the non-integrative nature of B-VEC, continuous application of the gel will be necessary to sustain the beneficial effects of the treatment.

In this study, we have combined the already established SMaRT technology with a minicircle MN511A1 vector to develop a non-viral therapeutic strategy to overcome the potential genotoxicity of integrating vectors [21]. The MN511A1 vector has already been used to successfully transfect RDEB keratinocytes with full-length *COL7A1* cDNA, leading to C7 expression in the monolayer [44]. With the SMaRT repair approach, we can leverage the benefits of a shorter transgene to be delivered, the maintenance of the endogenous regulation of the corrected protein and the correction of both recessively and dominantly inherited mutations. Recently, we have generated RTMs capable of replacing the complete 5′ half [22], as well as the 3′ half [21,45], of *COL7A1* upon their retroviral integration into the genome of RDEB keratinocytes.

Here, with our MC RTMS6m-expressing plasmid, we were able to transiently correct RDEB keratinocytes as well as fibroblasts in vitro on mRNA level and restore C7 expression in both. Due to observed divergences in transfection efficiencies with different reagents and methods depending on the cell type targeted, we used electroporation to introduce the RTM-expressing plasmids into RDEB fibroblasts and X-fect for the treatment of RDEB keratinocytes. With these treatment options, we generally achieved transfection efficiencies of up to 50% in RDEB fibroblasts and up to 30% in RDEB keratinocytes. For RTM delivery into 3D SEs, we obtained the most promising results by complexing the RTM-expressing plasmids with the liposomal carrier DDC642. Topical application of the RTM in combination with DDC642 liposomal carrier onto RDEB skin equivalents led to partial restoration of C7 expression, which was accurately localised to the basement membrane zone between the dermal and epidermal layers. Although the initial RTM treatment of patient-derived cells displayed a rather low correction efficiency at RNA and cellular level in monolayer culture, repeated RTM treatment (3 times per week, for 3 weeks) of SEs resulted in homogenous staining of C7 along the BMZ. While seemingly discordant with the results from monolayer treatment, in the context of skin tissue, C7 secreted into the extracellular space forms stabilised dimers that further assemble into supramolecular anchoring fibrils. The interaction of these anchoring fibrils with other extracellular matrix proteins of the BMZ, therefore, entraps and concentrates C7 within the BMZ. Given the long half-life of C7 of about 5 weeks, the homogenous C7 staining observed in RTM-treated SEs results from weeks-long continued deposition and accumulation of C7 along the BMZ. Accordingly, untreated RDEB-derived SEs also showed traces of mutated C7 accumulation within the BMZ, although C7 was not detected in the cytoplasm of these cells in a monolayer setting. Thus, even a low *trans*-splicing efficiency may be sufficient to provide adequate full-length C7 to restore skin integrity over time. However, this assumption has to be verified in appropriate animal models of RDEB skin, as the repair outcomes obtained in in vitro skin models are not directly translatable to the in vivo situation. In vivo, issues concerning application and delivery efficiencies in wounded or unwounded skin, in vivo stability of liposome-plasmid complexes, particularly within the context of a chronic wound, and the demonstration anchoring fibril restoration will need to be addressed in order to evaluate the true potential of RNA *trans*-splicing for in vivo RNA repair in RDEB. Currently, we are using the parental minicircle plasmid for our in vitro studies. As the size of the plasmid to deliver has a significant influence on its transfection efficiency in vitro, and especially in vivo, future studies in our established murine xenografting model will be performed with produced MCs as the size of the vector can be significantly reduced by about 4500 bp [46].

Since correction with our non-viral minicircle vector is not permanent, re-application of the complex would be necessary for further applications. However, the fact that C7 has a half-life of at least one month [47] makes a transient restoration of the protein at local target sites of the skin an attractive treatment option. Altogether, the data indicate that RNA *trans*-splicing may be a suitable in vivo treatment option for patients with dystrophic EB in the future.

## 4. Materials and Methods

### 4.1. Cell Culture & Cell Lines

RDEB keratinocytes were isolated from the hair follicles of a patient carrying a homozygous frameshift mutation (c.6081delC/c.6081delC) within exon 73 of *COL7A1*. RDEB fibroblasts were isolated from hair follicles carrying a mutation in exon 115 (c.8441-15del20/c.8505-8506dupCG) and from a phimosis carrying a mutation in exon 105 (c.7795G>T/c.7795G>T) (Table 1). Wild-type fibroblasts (WT Fib) and human keratinocytes (hKc) were isolated from a healthy donor upon informed consent. All cells were subsequently immortalised through transduction of the human papillomavirus proteins E6 and E7 and grown at 37 °C and 5% CO_2_ in a humidified incubator. Healthy keratinocytes, as well as patient-derived keratinocytes, were cultured in CnT-Prime Epithelial Proliferation Medium (CELLnTEC, Bern, Switzerland) with Primocin (InvivoGen, Toulouse, France) or in Gibco serum-free keratinocyte medium (SFM, Thermo Fisher Scientific, Waltham, MA, USA) supplemented with 100 U/mL penicillin/streptomycin (Biochrom, Berlin, Germany). Wild-type fibroblasts and RDEB fibroblasts were cultivated in CnT Fibroblast Medium (CELLnTEC, Bern, Switzerland) with primocin (InvivoGen, Toulouse, France).

### 4.2. Transfection and Electroporation of Keratinocytes and Fibroblasts

The RTMS6m carrying a binding domain specific for the intron 64/exon 65 junctions, was cloned into the parental minicircle MN511A1 plasmid (System Biosciences, Palo Alto, CA, USA) under the control of a cytomegalovirus (CMV) promoter, followed by an SV40 poly(A) signal.

Keratinocytes were grown to a confluency of 70–80% in 6-well plates and transfected with 5 to 7.5 µg plasmid DNA and 0.3 µL Xfect™ Transfection Reagent per µg plasmid DNA according to the manufacturer’s protocol (Takara Bio, Kusatu, Japan).

Fibroblasts were trypsinised and washed two times with PBS for nucleofection with the Neon™ Transfection System 10 μL Kit (Invitrogen, Waltham, MA, USA). 3 × 10^5^ cells were resuspended in buffer “R” provided in the Neon Transfection Kit, mixed with 900 ng of the plasmid and electroporated (1150 V, 30 ms, 2 pulses) with the Neon transfection system according to the manufacturer’s protocol (Thermo Fisher Scientific, Waltham, MA, USA). Treated cells were then seeded into antibiotic-free CnT-Prime (CELLnTEC, Bern, Switzerland) in 6-well plates. After 1 day of incubation, CnT-Prime with primocin (InvivoGen, Toulouse, France) was added, and cells were further cultured for either 48 h (RNA isolation) or 72 to 96 h (protein isolation, immunofluorescence staining).

### 4.3. RNA Isolation and SqRT-PCR

RNA from wild-type and RDEB cells were isolated 48 h hours after transfection with the innuPREP RNA Mini Kit (Analytik Jena, Jena, Germany). RNA was further transcribed into cDNA with the LunaScript RT SuperMix Kit (New England Biolabs, Ipswich, MA, USA) according to the manufacturer’s protocol. sqRT-PCR was performed with the Luna^®^ Universal qPCR Master Mix (New England Biolabs, Ipswich, MA, USA) on the CFX96 Touch Real-Time PCR Detection System (Bio-Rad, Hercules, CA, USA). GAPDH was used as a reference gene with a forward primer (5′-GCCAACGTGTCAGTGGTGGA-3′) and reverse primer (5′-CACCACCCTGTTGCTGTAGCC-3′). For detection of the *trans-*splicing reaction, a *COL7A1* exon 61/62 forward primer (5′-TGGGCCGAATGGTGCTGCA-3′) and an RTM-specific reverse primer (5′-CTGAATCTCCCT TTTCGCCCTTACG-3’) targeting the silent mutations introduced into the RTM was used. Cycling conditions were set at to 5 min at 95 °C, followed by 50 cycles of 30 s at 67 °C and 30 s at 72 °C, followed by a melt curve analysis spanning 67 °C to 95 °C. PCR products were analysed on a 2% agarose gel and confirmed by Sanger sequencing.

### 4.4. Next-Generation Sequencing

cDNA of treated cells and corresponding controls was amplified using the Phusion Polymerase at 67 °C with primers flanking the target region of exon 61/62 (5′-TCGTCGGCAGCGTCAGATGTGTATAAGAGACAGTGGGCCGAATGGTGCTGCA-3′) and exon 68, downstream of the silent mutations (5′-GTCTCGTGGGCTCGGAGATGTGTATAAGAGACAGCTGGAGGCCCCTGGGGTCCA-3′). NGS preparation was performed as recently described [36]. Illumina MiSeq Nano PE250 sequencing of amplicons was performed by the Vienna Biocenter Next-Generation Sequencing Facility (VBCF, Vienna, Austria). Analysis of the NGS was performed with CRISPresso2 (http://crispresso.pinellolab.org/submission) (accessed on 25 November 2022) [25].

### 4.5. Immunofluorescence Staining of C7 in Keratinocytes and Fibroblasts

72 to 96 h after transfection, 5 × 10^4^ cells were seeded into chamber slides and fixed with 4% Para-formaldehyde for 30 min at RT upon reaching confluency. After three washing steps with 1× PBS, cells were incubated with a 1× Blocking buffer (Roche Diagnostics GmbH, Mannheim, Germany) in PBS at RT for 1 h. Type VII collagen was detected with an anti-collagen type VII rabbit polyclonal antibody [26] diluted 1:2000 in PBS with 0.3% Triton X-100 for 4 h at room temperature. Alexa-Fluor-488 goat anti-rabbit (Thermo Fisher Scientific, Waltham, MA, USA) (1:400 in PBS) was used as a second antibody for 1 h in combination with DAPI (1:2000) (VWR, Vienna, Austria). Cells were stored in PBS and analysed using the confocal laser scanning microscope Axio Observer Z1 attached to LSM700 (Carl Zeiss, Berlin, Germany).

### 4.6. Protein Isolation and Western Blot Analysis

For protein isolation, cells were lysed with a radioimmunoprecipitation assay (RIPA) buffer (Santa Cruz Biotechnology, Heidelberg, Germany) 72 to 96 h after transfection. After lysis, cells were centrifuged at 350× *g* for 5 min at 4 °C, and the clarified supernatant was then frozen at −20 °C. For loading onto an 8% BisTris-Gel, proteins were mixed with a 4× loading buffer (0.25 M tris, 8% SGS, 30% glycerol, 0.02% bromphenol blue [pH 6.8]) and denatured at 95 °C for 5 min. Western blot analysis was performed as previously described [32]. The nitrocellulose membrane was blocked with 10× blocking reagent from Roche Diagnostics (Roche Diagnostics GmbH, Mannheim, Germany) diluted 1:10 in Tris-buffered saline with 0.2% Tween (TBS-T) for 1 h at room temperature. C7 was detected via a polyclonal anti-C7 antibody [26] at a dilution of 1:1000 in TBS-T. Antibodies against α-actinin (H-300 sc-15335; Santa Cruz, Dallas, TX, USA) or β-tubulin (ab6064; Abcam, Cambridge, UK) were used as loading controls in a dilution of 1:1000 (α-actinin) or 1:2000 (β-tubulin). The membrane was incubated overnight at 4 °C with the primary antibody. A goat anti-rabbit HRP-labelled antibody (Dako, Santa Clara, CA, USA) was used as a secondary antibody. The membrane was incubated for 1 h at a dilution of 1:400 in TBS-T. Protein bands were visualised with the Immobilon Western Chemiluminescent HRP Substrate (Merck, Darmstadt, Germany) and the ChemiDoc XRS Imager (BioRad, Hercules, CA, USA). Densitometric analysis of band intensities was performed using the Image Lab™ Software from BioRad (BioRad, Hercules, CA, USA).

### 4.7. Generation and Transfection of Skin Equivalents 

A human fibrin scaffold was used for the generation of skin equivalents. 5 × 10^4^ fibroblasts were immersed in a fibrinogen scaffold consisting of DMEM with 20% FCS, fibrinogen (F4883; Sigma-Aldrich, St. Louis, MO, USA) in 0.9% NaCl (final concentration = 25 mg/mL), thrombin (T8885; Sigma-Aldrich, St. Louis, MO, USA) dissolved in 25 mM CaCl_2_ and aprotinin (A6279; Sigma-Aldrich, St. Louis, MO, USA). The scaffold was directly prepared in Falcon^®^ permeable support inserts with a 0.4 µm transparent PET membrane (Corning, New York, NY, USA) and placed in BioCoat™ Deep-Well Plates (6-well, Corning, New York, USA) for 1 h at 37 °C and 5% CO_2_. 1 × 10^5^ keratinocytes per well were seeded on top of the matrix and grown to confluence in DMEM:Ham’s F-12 Green’s keratinocyte medium. Skin equivalents were then raised to an air–liquid interface and cultured for 21 days to allow stratification before they were treated with the DDC642 liposome/MC RTMS6m complex.

For the transfection of the SE, 5 µg of the plasmid was complexed in a ratio of 1:8 with DDC642 liposomes (40 µg) by adding the liposomes to the plasmid under continuous vortexing. After 10 min of incubation at room temperature, the complex was topically applied to the skin’s surface three times over the course of one week. Skin equivalents were harvested approximately 72 to 96 h after the last treatment.

### 4.8. Generation of DDC642 Liposomes

For the generation of DDC642 liposomes [29], DOTAP (Sigma-Aldrich, St. Louis, MO, USA), DOPE (Sigma-Aldrich, St. Louis, MO, USA) and cholesterol (Sigma-Aldrich, St. Louis, MO, USA) were dissolved in chloroform (Sigma-Aldrich, St. Louis, MO, USA) and mixed in a ratio of 6 to 4.2 to 1.8. Chloroform was evaporated at 37 °C for approx. 4 h, resuspended with 1 mL 30% EtOH-HEPES and incubated at room temperature in the dark for one day. The mixture was repeatedly extruded through a 100 nm polycarbonate membrane using a LiposoFast Factory (Sigma-Aldrich, St. Louis, MO, USA) and then stored at 4 °C.

### 4.9. Immunofluorescence Staining of Skin Equivalents

Cryosections of 8 µm were fixed with acetone:methanol (1:1) at −20 °C for approx. 20 min and washed with PBS twice for 5 min. The slides were then incubated for 4 h with a human-specific anti-C7 antibody [26] diluted 1:2000 in 1× blocking reagent (Roche Diagnostics GmbH, Mannheim, Germany) in PBS with 0.2% Tween and 0.3% Triton X-100 (PBSTX). After three washing steps with PBS, visualisation of the staining was performed using Alexa Fluor 488 goat anti-rabbit IgG (H + L) secondary antibody (Thermo Fisher Scientific, Waltham, MA, USA) diluted 1:400 and DAPI (4′,6-Diamidin-2-phenylindol) (Thermo Fisher Scientific, Waltham, MA, USA) diluted 1:2000 in PBS for 1 h at room temperature. After three washing steps with PBS for 5 min, cryosections are covered with a DAKO fluorescent mounting medium (Agilent, Santa Clara, CA, USA). Skin sections were analysed using the confocal laser scanning microscope Axio Observer Z1 attached to LSM700 (Carl Zeiss, Berlin, Germany).

The mean fluorescence intensity of raw image files (Carl Zeiss Image, CZI) was analysed using Fiji [27]. Final MFI calculations were done by subtracting the background expression from the sample MFI [28]. Statistical analyses were performed using GraphPad Prism 9 (GraphPad Software, La Jolla, CA, USA) for unpaired Student’s *t*-tests.

### 4.10. Immunohistochemistry Staining of Skin Equivalents 

Cryosections were also used for hematoxylin and eosin stainings, where hematoxylin stains the cell nuclei and eosin stains the extracellular matrix and cytoplasm. After 30 washing steps with H_2_O, slides were re-incubated for 6 min in Mayer’s hemalum solution (Merck, Kenilworth, NJ, USA), followed by another 30 washing steps in H_2_O. The cryosections are dipped ten times in a 0.3% HCl/EtOH solution, 30 times in H_2_O, and further incubated for 2 min in a 0.5% Eosin G solution (Merck, Kenilworth, NJ, USA). Slides are then washed 30 times in H_2_O, 30 times in Isopropanol and 30 times in HistoChoice^®^ Clearing Agent (Merck, Kenilworth, NJ, USA). Finally, cryosections are covered with ROTI^®^Histokitt (Carl Roth, Karlsruhe, Germany) and stored at 4 °C.

## 5. Patents

Johann W. Bauer is an inventor on a patent on Improved pre-mRNA trans-splicing molecule (RTM) molecules and their uses. EU: 1402/2320952; US: 8,735,366; Japan: 5735912.

## Figures and Tables

**Table 1:** RDEB cell lines.

Cell Line	Mutation	Exon	Cell Type
RDEB1	c.6081delC/c.6081delC	exon 73	keratinocytes
RDEB2	c.8441-15del20/c.8505-8506dupCG	exon 115	fibroblasts
RDEB3	c.7795G>T/c.7795G>T	exon 105	fibroblasts

## Data Availability

All necessary datasets, including NGS data, can be found in the Zenodo database: https://zenodo.org/record/7403098.
